# When the Medium Skews the Message: Commercial Mueller–Hinton Agar Media and Their Effect on Antibiogram Results of Imipenem, Ciprofloxacin, and Gentamicin Against Standard and Clinical Strains of *Pseudomonas aeruginosa*

**DOI:** 10.1155/ijm/1638304

**Published:** 2026-11-09

**Authors:** Amir Reza Abdollahi, Seyed Sadeq Mousavi Ghahfarrokhi, Fatemeh Aghajafari, Hossein Jafari-Nodoushan, Marjan Tootianquds, Nasrin Samadi

**Affiliations:** ^1^ Department of Drug and Food Control Faculty of Pharmacy Tehran University of Medical Sciences Tehran Iran tums.ac.ir; ^2^ Research Group for Quality Control and Development of Pharmaceutical Food and Cosmeticeutical Products The Institute of Pharmaceutical Sciences (TIPS) Tehran University of Medical Sciences Tehran Iran tums.ac.ir; ^3^ Universal Scientific Education and Research Network (USERN) Tehran Iran usern.org; ^4^ Department of Drug and Food Control Faculty of Pharmacy and Biotechnology Research Center International Campus Tehran University of Medical Sciences (IC-TUMS) Tehran Iran; ^5^ Department of Pharmaceutics School of Pharmacy Shahid Sadoughi University of Medical Sciences and Health Services Yazd Iran ssu.ac.ir; ^6^ Diamond New Age Sağlık Gıda Turizm Sanayi Ticaret İthalat İhracat Limited Şirketi Ankara Turkey

**Keywords:** antibiogram disk, ciprofloxacin, gentamicin, imipenem, Mueller–Hinton agar, *Pseudomonas aeruginosa*

## Abstract

Accurate antimicrobial susceptibility testing (AST) is essential for guiding effective therapy and monitoring antibiotic resistance, particularly for *Pseudomonas aeruginosa*, a pathogen noted for its intrinsic and adaptive resistance mechanisms. Mueller–Hinton agar (MHA) is the standard medium for AST; however, variations in its composition among commercial brands can significantly affect test outcomes. This study evaluated the influence of five different commercial MHAs (HiMedia, Merck, Condalab, Quelab, and Sigma) on the antibiogram results of imipenem, ciprofloxacin, and gentamicin against both standard (*P. aeruginosa* ATCC 15442) and 50 clinical isolates. Three inoculation methods, swab, pour plate, and double‐layer, were compared with identify the most reproducible approach. Inductively coupled plasma mass spectrometry (ICP‐MS) was used to quantify divalent cations (Ca^2+^, Mg^2+^, Zn^2+^, Mn^2+^, Fe^2+^) in each medium, and microscopic examination assessed gel microstructure. The double‐layer method demonstrated the lowest coefficient of variation, indicating superior reproducibility compared with the swab and pour plate techniques. Significant differences were observed among media in inhibition zone diameters, with Quelab and Condalab producing consistently smaller zones (**p** < 0.05). Cation analysis revealed wide variability, with calcium and magnesium levels differing up to fourfold across brands and being associated with differences in antibiotic activity, particularly for gentamicin and ciprofloxacin. Microscopic evaluation revealed minor gel‐structure variations without measurable impact on antibiotic diffusion. Susceptibility profiles also differed among MHA brands. These findings highlight the potential importance of medium composition, particularly cation concentration, in the variability of AST results. Standardization and rigorous quality control of commercial MHAs are essential to ensure reliable, reproducible, and clinically meaningful susceptibility testing.

## 1. Introduction

*Pseudomonas aeruginosa* is a gram‐negative bacterium widely recognized as a significant opportunistic pathogen. Although it rarely causes disease in healthy individuals, it presents a major threat to immunocompromised patients, such as those with cystic fibrosis, cancer, AIDS, or severe burns, and individuals with implanted medical devices [1, 2]. Its adaptability to diverse environments—including soil, water, and hospital surfaces—contributes to its prevalence and clinical relevance, particularly in healthcare settings where it is a leading cause of hospital‐acquired infections such as ventilator‐associated pneumonia, catheter‐associated urinary tract infections, bloodstream infections, and surgical site infections [3, 1]. Antimicrobial susceptibility testing (AST) plays a crucial role in managing infections caused by *P. aeruginosa*, a pathogen known for its intrinsic and adaptive resistance mechanisms [4]. AST allows clinicians to determine which antibiotics are likely to be effective against a specific bacterial isolate, enabling targeted therapy that improves patient outcomes and limits the spread of resistance. Without AST, empirical treatment can be unreliable due to the organism′s rapidly changing resistance patterns. In addition, AST supports antimicrobial stewardship by minimizing unnecessary use of broad‐spectrum antibiotics, which helps prevent the development of multidrug‐resistant strains [5].

Antibiograms, which summarize the susceptibility of local bacterial isolates over time, are valuable tools for guiding initial antibiotic choices before AST results are available. However, their reliability decreases with prolonged hospitalization as resistance can evolve, particularly affecting drugs like imipenem and ceftazidime [5, 6]. Commonly used antibiotics against *P. aeruginosa* include imipenem, ciprofloxacin, and gentamicin, each with susceptibility rates around 74%–85%, though resistance mechanisms vary. Other effective options include piperacillin‐tazobactam, ceftazidime‐avibactam, amikacin, and newer agents like ceftolozane/tazobactam and cefiderocol, which are designed to combat resistant strains [7–9].

Mueller–Hinton agar (MHA) is widely regarded as the gold standard medium for AST, especially in the disk diffusion (Kirby–Bauer) method [10, 11]. It is endorsed by both the Clinical and Laboratory Standards Institute (CLSI) and the European Committee on Antimicrobial Susceptibility Testing (EUCAST) for use with nonfastidious bacteria like *P. aeruginosa* [12]. MHA′s popularity is due to its consistent ability to support the growth of clinically important bacteria and its reliable, well‐characterized performance [13]. To ensure accuracy and reproducibility in test results, commercially prepared MHA intended for AST is subject to quality‐control requirements, with each batch undergoing verification using reference bacterial strains to confirm that inhibition zones fall within acceptable ranges.

Several compositional and physical factors in MHA can significantly influence AST outcomes if not tightly controlled. For instance, calcium and magnesium ion concentrations affect the efficacy of certain antibiotics such as aminoglycosides, while pH levels can alter the activity of drugs like quinolones and sulfonamides [14, 15]. Nutrient variations, thymidine or thymine content, agar depth, and batch‐to‐batch inconsistencies can also impact bacterial growth and antibiotic diffusion, leading to inaccurate susceptibility interpretations [16, 17]. To minimize such variability, modern MHA formulations are produced according to ISO standards, with precise control over pH, cation content, and other key parameters. Rigorous quality control ensures reliable, consistent results across laboratories and manufacturers worldwide.

Although MHA is considered the gold standard for AST, studies have revealed significant variability among different commercial brands and even between production lots from the same manufacturer [13, 18]. This inconsistency can lead to differences in inhibition zone diameters and minimum inhibitory concentration (MIC) values, which affects the reliability of AST results. For example, testing *P. aeruginosa* with gentamicin or polymyxin B on different MHA lots has shown substantial variation in outcomes, often due to fluctuating cation concentrations like calcium, magnesium, and zinc [14, 15, 19]. Variability among different brands and lots of MHA can also significantly influence the results of AST, particularly for antibiotics like cefiderocol used against resistant gram‐negative bacteria [18]. Studies have shown that discrepancies in MHA quality can lead to inconsistent interpretations in disk diffusion testing, emphasizing the need for harmonization of testing standards and breakpoint criteria across regulatory bodies. Factors such as agar depth—specified by EUCAST to be 4 ± 0.5 mm—also affect AST accuracy [20]. Quality control is therefore critical, as demonstrated by studies showing that variations in MHA can alter susceptibility results for drugs like gentamicin against *P. aeruginosa*, with results closely linked to the behavior of reference strains such as *P. aeruginosa* ATCC 27853. These inconsistencies can compromise the clinical interpretation of susceptibility data, potentially leading to inappropriate antibiotic selection. The underlying cause of this variability lies in inconsistent manufacturing practices, including differences in raw material sourcing, nutrient content, ion concentration, pH control, and adherence to quality standards. Although international guidelines from bodies like CLSI and EUCAST call for strict quality control, not all manufacturers meet these expectations consistently. Some improve only after external evaluation highlights deficiencies. As a result, discrepancies in MHA quality can affect patient care by producing misleading AST results and potentially contributing to the development and spread of antimicrobial resistance. The findings underscore the critical need for standardized production protocols and rigorous quality assurance across all MHA products.

In particular, the novelty and aim of this study lie in the integrated evaluation of five commercially available MHA brands in AST of *P. aeruginosa*, combining (i) comparison of inhibition‐zone diameters for clinically relevant antibiotics, (ii) evaluation of three different inoculation techniques, (iii) quantitative determination of divalent and trace‐element cation concentrations using ICP‐MS, and (iv) microscopic assessment of the agar microstructure. Importantly, the study also evaluates these effects using a relatively large collection of 50 clinical *P. aeruginosa* isolates in addition to a standard strain. This integrated approach allows us to examine potential relationships between physicochemical variability of commercial MHA and variability in antimicrobial susceptibility results.

## 2. Materials and Methods

MHA was obtained from HiMedia Laboratories Pvt. Ltd., India; Merck KGaA, Germany; Condalab, Spain; Quelab Laboratories Inc., Canada; and Sigma‐Aldrich, United States. Antibiotic disks containing ciprofloxacin, gentamicin, and imipenem were obtained from Mast Group Ltd., United Kingdom, and Padtan‐Teb Co., Iran. Cetrimide agar was obtained from HiMedia Laboratories Pvt. Ltd., India. N,N,N ^′^,N ^′^‐tetramethyl‐p‐phenylenediamine dihydrochloride was obtained from HiMedia Laboratories Pvt. Ltd., India. The testing was conducted using MHA obtained from five different commercial manufacturers. To achieve this objective, the following procedures were carried out:

### 2.1. Sampling, Isolation, and Identification of Clinical *P. aeruginosa* Strains

Fifty clinical isolates of *P. aeruginosa* were obtained from the microbiology laboratory of the participating hospital. The isolates were subsequently recultured and processed in our microbiology laboratory before being used for AST. The isolates were provided to the research laboratory without individual patient‐level clinical metadata.

After colony growth, the isolates were purified by quadrant streaking on cetrimide agar and incubated again at 37°C for 24 h. Gram staining was performed to identify gram‐negative, rod‐shaped, pink‐stained bacteria for further analysis. Then, the oxidase test was conducted by applying a 1% tetramethyl‐p‐phenylenediamine dihydrochloride reagent on filter paper [21]. The combination of these methods ensured accurate isolation and identification of *P. aeruginosa* from clinical samples.

### 2.2. Growth Promotion Test

Growth promotion testing was performed on five commercial MHA media (HiMedia, Merck, Condalab, Quelab, and Sigma Co.) to evaluate their ability to support the growth of the test microorganism. P of 20 mL from each molten MHA medium was inoculated with 1 mL of a standardized microbial suspension of *P. aeruginosa* ATCC 15442 containing about 10^2^ CFU/mL in Petri dishes. The plates were then gently rotated on a flat surface using a figure‐eight motion to ensure thorough mixing of the inoculum with the medium. The plates were allowed to solidify and incubated at 37°C for 18–24 h. After incubation the number of grown colonies in each medium was counted. The nutritive properties of the media were considered acceptable if the number of recovered colonies did not differ by more than a factor of two from the calculated inoculum.

### 2.3. Assessment of Antibiotic Disks Performance by Standard *P. aeruginosa* Strain

Prior to testing the clinical isolates, the performance of the antibiotic disks used in the study (from Mast Co. and Padtan‐Teb Co.) was verified using *P. aeruginosa* ATCC 15442 as a reference strain. This assessment was performed to verify the performance of the prepared antibiotic disks under the experimental conditions. The clinical isolates were subsequently tested using the validated disk diffusion method, and their inhibition‐zone diameters were interpreted according to the CLSI clinical breakpoints for *P. aeruginosa*.

For this study, MHA plates were prepared according to the CLSI guideline [12]. For each type of culture medium, the entire study was conducted using a single lot obtained at the beginning of the study. No different lots of the same medium were used during the experiments. A precisely measured volume of 25 mL of sterile molten MHA was dispensed into each 90‐mm Petri dish using a sterile 25‐mL serological pipette and pipette controller, resulting in a target agar depth of approximately 4 mm. The agar volume was kept constant across all plates to ensure consistent agar depth. The microbial inoculum was calibrated by establishing a correlation between optical density at 600 nm (OD600) and viable bacterial counts (CFU/mL) using the pour‐plate method. An OD600 value corresponding to approximately 1.5 × 10^8^ CFU/mL was determined and used for inoculum standardization. Prior to each experiment, a fresh microbial suspension was prepared and adjusted to the predetermined OD600 value corresponding to approximately 1.5 × 10^8^ CFU/mL. The suspension was uniformly inoculated onto the agar surface using a sterile swab. Then, antibiotic disks were placed on the agar surface, maintaining a distance of 25 mm between disk centers and 16 mm from the edge of the plate. The inoculated plates were then incubated under aerobic conditions at 36.5^°^C ± 0.5^°^C for 22‐24 h. Following incubation, the diameters of the growth inhibition zones were measured using a caliper [12]. Each experiment was conducted using three independent biological replicates, with three technical replicates for each biological replicate.

### 2.4. Selection of Disk Diffusion Method

Three inoculation techniques, swab, pour plate, and double‐layer, were compared using the five commercial MHA brands and the tested antibiotic disks. The procedures are described below.

#### 2.4.1. Swab Inoculation Method

Initially, 25 mL of each of the five sterile MHA media was aseptically dispensed into sterile Petri dishes. The plates were allowed to solidify with the lids partially open. Once solidified, the prepared microbial suspension (1.5 × 10^8^ CFU/mL) was uniformly spread over the entire surface of each plate using a sterile swab. After allowing the surface to dry, antibiotic disks were applied. In this study, disks were placed in triplicate—three of each type and brand. Following disk placement, the plates were sealed and incubated at 37°C for 20–22 h.

#### 2.4.2. Pour Plate Method

In this method, the molten MHA medium, previously inoculated with a 1% microbial suspension at a concentration of about 10^8^ CFU/mL, was divided into 25 mL volumes in Petri dishes [22]. The plates were allowed to solidify and dry, after which antibiotic disks were applied for susceptibility testing.

#### 2.4.3. Double‐Layer Method

As the name implies, this method involves the application of the MHA medium in two distinct layers. The first layer consists of 20 mL of MHA medium, which is poured into the Petri dish and allowed to solidify under aseptic conditions before the addition of the second layer. For the second layer, the MHA medium, previously inoculated with a 1% microbial suspension at a concentration of about 10^8^ CFU/mL, was divided into a volume of 5 mL on the solidified first layer. Once the second layer has solidified and dried, antibiotic disks were applied to the surface of the plates for subsequent susceptibility testing.

### 2.5. Quantification of Cation Concentrations in Culture Media

The concentrations of calcium (Ca), magnesium (Mg), zinc (Zn), manganese (Mn), and iron (Fe) ions in the aforementioned culture media were determined using inductively coupled plasma mass spectrometry (ICP‐MS). Subsequently, the relationship between the measured cation concentrations and the observed variability in antibiotic susceptibility results was examined.

### 2.6. Examination of Agar Culture Media Capillary Spaces

To observe the agar culture media capillary spaces, a thin layer, approximately 1 mm in diameter, was carefully removed from each MHA medium and examined using an Epi fluorescence microscope (Leica DMI 600 B) equipped with a 100× objective lens.

### 2.7. Antibiotic Resistance Pattern of Clinical *P. aeruginosa* Strains

Based on the results obtained, the double‐layer method was selected for further investigation of the antibiotic susceptibility of clinical strains. As detailed in the methodology section, the antibiotic resistance pattern of *P. aeruginosa* isolates was assessed using the disk diffusion method (Kirby–Bauer) in accordance with CLSI guidelines (Table 1). The susceptibility of these strains to three commonly used antibiotics, gentamicin, ciprofloxacin, and imipenem, was evaluated by comparing antibiotic disks from Mast Co. and Padtan Teb Co. under controlled laboratory conditions. This method was applied to determine the sensitivity profiles of all 50 clinical isolates by using MHA media from five different companies, HiMedia, Merck, Condalab, Quelab, and Sigma. The disk diffusion assay was performed according to the relevant CLSI guidelines. In addition, the appearance of one of the clinical strains in culture medium was examined in terms of pigment production in these five culture media.

**Table 1:** Zone diameter breakpoints for interpretation of antimicrobial susceptibility of *P. aeruginosa* in response to ciprofloxacin, gentamicin, and imipenem disks based on CLSI interpretive standards [12].

Antibiotic	Disk content	Inhibition zone (mm)		
		Sensitive	Intermediate	Resistance
**Ciprofloxacin**	**5 *μ*g**	**25**	**19–24**	**18**
**Gentamicin**	**10 *μ*g**	**15**	**13–14**	**12**
**Imipenem**	**10 *μ*g**	**19**	**16–18**	**15**

### 2.8. Statistical Analysis

All experiments were performed in triplicate, and data are presented as the mean ± standard deviation (SD). One‐way analysis of variance (ANOVA) was conducted using SigmaPlot software (Version 16.0), with **p** < 0.05 considered statistically significant. The coefficient of variation (CV (%)) was calculated as the percentage ratio of the SD to the mean and used as an indicator of method reproducibility.

## 3. Results

### 3.1. Growth Promotion Test

The growth‐promotion test results, conducted in three replicates per day over three consecutive days, indicated that the MHA media had acceptable nutritional properties and growth promoting potential because the number of recovered colonies did not differ by more than a factor of two from the expected value for the standardized inoculum. The data revealed no microbiological differences in colony counts among the five commercial growth media tested (Table 2).

**Table 2:** Growth promotion test results of five commercial brands of Mueller–Hinton agar by using *Pseudomonas aeruginosa* ATCC 15442, conducted in three replicates per day over three consecutive days.

Day	Colony per plate				
	HiMedia	Merck	Condalab	Quelab	Sigma
**1**	90.67 ± 3.51^**a**^	89.33 ± 2.08	102.33 ± 3.06	91.33 ± 4.16	100.67 ± 6.66
**2**	83.67 ± 4.73	102.33 ± 3.06	86.67 ± 2.08	93.33 ± 5.68	88.00 ± 4.58
**3**	94.67 ± 4.16	83.67 ± 3.79	91.00 ± 1.73	86.33 ± 5.86	90.67 ± 5.03

^a^Mean ± SD.

### 3.2. Assessment of Antibiotic Disks Performance by Using Standard *P. aeruginosa* Strain

The average diameter of the growth inhibition zones for each antibiotic, irrespective of the culture method or brand of culture medium used, consistently fell within the CLSI‐specified acceptable ranges (> 25, 15, 19 mm for ciprofloxacin, gentamicin, and imipenem, respectively) [23]. This assessment provides evidence of the performance of the antibiotic disks under the experimental conditions used in this study.

### 3.3. Selection of Disk Diffusion Method

The impact of inoculation technique, brand of MHA medium, and antibiotic disk were systematically investigated. The following variables were included: MHA medium: five brands “HiMedia, Merck, Condalab, Quelab, and Sigma;” antibiotics tested: ciprofloxacin, gentamicin, and imipenem; antibiotic disks: two brands, Mast (United Kingdom) and Padtan‐Teb (Iran); inoculation techniques: swabbing, pour plate, and double layer methods. The disk diffusion test was conducted using all three inoculation techniques The goal was to assess how these variables influenced bacterial sensitivity to the antibiotics, as assessed by inhibition‐zone diameter. To ensure reliability, each antibiotic disk was tested in triplicate on each culture medium. The results, reported as the SD of the inhibition zone diameters, are presented in Table 3.

**Table 3:** Summary of disk diffusion test results evaluating three inoculation techniques—swabbing, pour plate, and double‐layer methods—on the standard strain of *P. aeruginosa* using three antibiotics (ciprofloxacin, gentamicin, and imipenem) across five different commercial brands of MHA.

**Inhibition zone diameter (mm)/Mast Co.**							
**Cultivation technique**	**Antibiotic**	**HiMedia**	**Merck**	**Condalab**	**Quelab**	**Sigma**	**CV (%)**

**Swab**	**Ciprofloxacin**	24.49 ± 1.58	25.03 ± 4.57	24.38 ± 2.29	22.88 ± 3.10	23.81 ± 4.85	**19.17**
	**Gentamicin**	20.96 ± 1.98	20.93 ± 1.74	18.74 ± 3.84	16.23 ± 4.29	19.99 ± 4.61	
	**Imipenem**	25.21 ± 1.44	25.58 ± 2.32	21.10 ± 4.70	15.53 ± 2.10	21.72 ± 3.26	
**Pour plate**	**Ciprofloxacin**	22.32 ± 2.32	23.63 ± 4.43	22.67 ± 1.62	23.49 ± 1.14	22.82 ± 3.24	**14.13**
	**Gentamicin**	18.93 ± 2.39	19.10 ± 0.82	18.73 ± 0.43	16.49 ± 0.29	18.29 ± 1.48	
	**Imipenem**	21.66 ± 0.50	23.92 ± 1.71	19.78 ± 0.86	17.29 ± 2.75	22.10 ± 0.24	
**Double layer**	**Ciprofloxacin**	23.84 ± 1.50	23.38 ± 3.08	24.28 ± 5.61	24.04 ± 0.71	22.94 ± 3.20	**13.63**
	**Gentamicin**	21.12 ± 2.80	21.09 ± 1.12	20.11 ± 0.18	16.78 ± 0.47	19.88 ± 1.55	
	**Imipenem**	23.53 ± 1.28	24.92 ± 1.14	21.47 ± 2.27	18.81 ± 2.35	23.55 ± 0.57	

**Inhibition zone diameter (mm)/Padtan-Teb Co.**							
**Cultivation technique**	**Antibiotic**	**HiMedia**	**Merck**	**Condalab**	**Quelab**	**Sigma**	**CV (%)**

**Swab**	**Ciprofloxacin**	24.17 ± 2.23	26.26 ± 4.34	26.20 ± 3.52	22.73 ± 2.52	23.76 ± 5.13	**28.97**
	**Gentamicin**	19.51 ± 2.08	20.01 ± 1.81	16.90 ± 3.70	14.59 ± 4.87	18.19 ± 4.71	
	**Imipenem**	16.77 ± 1.56	21.56 ± 4.19	11.27 ± 1.24	12.62 ± 2.05	12.81 ± 0.65	
**Pour plate**	**Ciprofloxacin**	21.93 ± 1.94	22.75 ± 3.43	22.99 ± 2.17	15.54 ± 13.62	24.55 ± 4.06	**28.94**
	**Gentamicin**	17.53 ± 2.46	18.52 ± 1.38	18.77 ± 1.82	15.15 ± 0.74	17.04 ± 1.23	
	**Imipenem**	16.29 ± 3.57	19.84 ± 6.80	15.88 ± 6.79	13.16 ± 4.74	16.04 ± 4.92	
**Double layer**	**Ciprofloxacin**	23.46 ± 1.93	23.19 ± 3.01	24.60 ± 5.60	25.44 ± 2.24	22.72 ± 3.27	**26.24**
	**Gentamicin**	20.43 ± 2.19	20.90 ± 1.02	19.68 ± 0.80	15.98 ± 0.61	19.48 ± 0.83	
	**Imipenem**	19.93 ± 5.44	17.75 ± 6.33	15.40 ± 7.53	12.94 ± 7.44	14.80 ± 6.59	

As illustrated in Table 3, CV (%) for all three inoculation methods is presented. Based on the comparative analysis of these graphs, the double‐layer method produced the lowest CV among the three inoculation techniques, indicating the highest reproducibility under the conditions evaluated. Accordingly, this method was selected for subsequent testing of the clinical isolates.

The overall CV for Mast antibiotic disks was significantly lower than that of Padtan‐Teb, indicating greater precision and consistency. As shown in Table 3, among the tested inoculation techniques, the swabbing method exhibited the highest CV, reflecting lower precision. In contrast, the pour plate and double‐layer methods demonstrated comparable and lower CVs, suggesting higher reliability and reproducibility in those approaches.

Statistical analysis using ANOVA revealed significant differences among various culture media in the formation of growth inhibition zones. Specifically, Quelab and Condalab media consistently exhibited the smallest inhibition zones against *P. aeruginosa* when tested with different antibiotic disks (Mast, Padtan‐Teb) and techniques (swabbing, pour plate, and double‐layer). The results illustrated in Figure 1, were statistically significant (**p** < 0.05), indicating that the choice of culture medium notably influences the observed efficacy of antibiotics such as imipenem and gentamicin.

**Figure 1:**
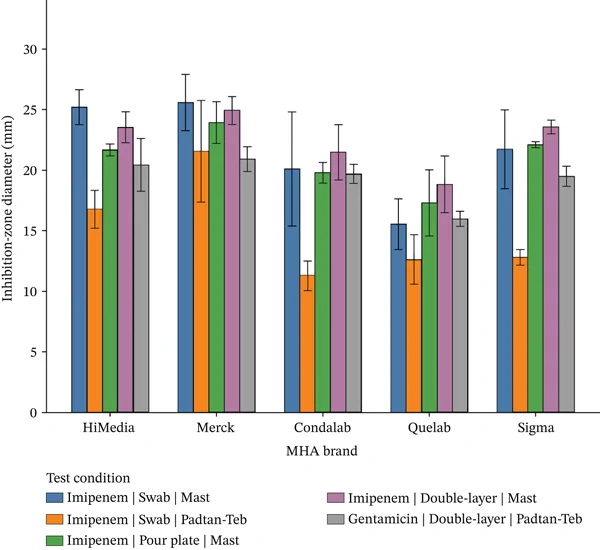
Effect of culture media brand and diffusion method on inhibition zones of antibiotic disks against *P. aeruginosa* ATCC 15442. Error bars: mean ± SD. One‐way ANOVA across MHA brands with each text condition; all comparisons were statistically significant (*p* < 0.05).

Given the results obtained and the higher reproducibility demonstrated by the double‐layer method, this technique was selected for subsequent stages of the study.

Direct breakpoint crossing was most pronounced with Padtan‐Teb imipenem disks using the standard swabbing technique. On Merck agar, the isolate maintained a susceptible classification shifted to intermediate on HiMedia, and was categorized as resistant on Sigma, QueLab, and Condalab. Inoculation methodology substantially influenced variance across agar brands. The standard swab method exhibited high inter‐brand coefficient of variation. In contrast, adopting the double‐layer technique mitigated brand‐dependent dispersion, dropping total CV to 12.04% for imipenem (Mast) and 10.75% for gentamicin (Padtan‐Teb), while maintaining susceptibility categorization on Merck, HiMedia, Condalab, and Sigma.

### 3.4. Cation Concentration in Culture Media

The concentrations of calcium (Ca), magnesium (Mg), zinc (Zn), manganese (Mn), and iron (Fe) in the tested culture media were determined using ICP‐MS. The results are presented in Table 4.

**Table 4:** The concentration (ppm) of aluminum, calcium, iron, magnesium, manganese, and zinc ions in the five brands of Mueller–Hinton agar medium.

Cation	HiMedia	Merck	Condalab	Quelab	Sigma
**Al**	**50.9**	**15.4**	**13.7**	**21.8**	**14.7**
**Ca**	**90.3**	**122.4**	**421.1**	**347.5**	**226.2**
**Fe**	**113.5**	**90.2**	**102.7**	**122.8**	**96.7**
**Mg**	**156.2**	**62.0**	**57.5**	**195.2**	**164.1**
**Mn**	**2.3**	**2.7**	**2.1**	**4.1**	**1.8**
**Zn**	**4.36**	**5.28**	**6.62**	**8.28**	**5.89**

### 3.5. Agar Culture Media Capillary Spaces

The morphology and dimensions of the capillary spaces formed within the gels of different MHA media vary and range from about 0.3–2.9 *μ*m as shown in Figure 2. These microstructures are occupied by water molecules. Considering that all antibiotic molecules used in this study possess molecular weights of below 500 Daltons, corresponding to sizes under 1 nm [24], therefore, the size of these capillary spaces may not impose significant restrictions on their diffusion. Consequently, the distribution of antibiotics within the gel matrix remains consistent across different brands of culture media. However, because diffusion coefficients or diffusion kinetics were not directly measured, the present microscopic observations do not allow a quantitative assessment of the effect of capillary‐space dimensions on antibiotic diffusion.

**Figure 2:**
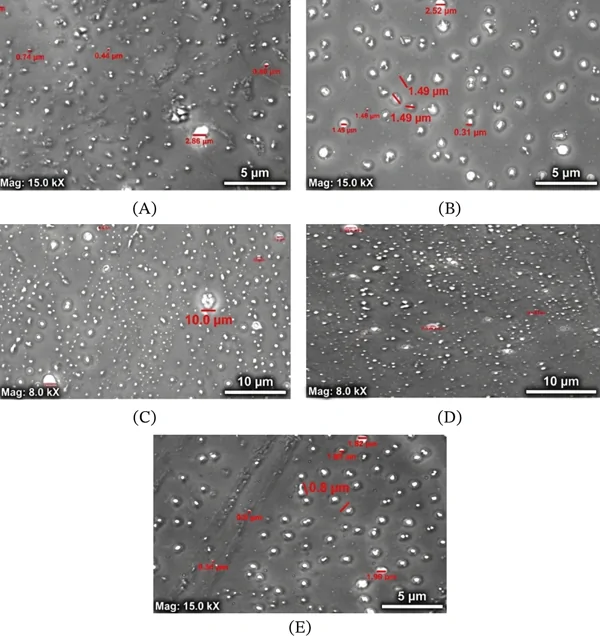
The morphology and dimensions of the capillary spaces formed within the gels (A) HiMedia, (B) Merck, (C) Condalab, (D) Quelab, and (E) Sigma.

### 3.6. Antibiotic Resistance Pattern of Clinical *P. aeruginosa* Strains

The diameter of growth inhibition zones for the antibiotics tested in this study, using disk diffusion method, was interpreted according to the CLSI interpretive zones as indicated in Table 1 [12].

The antibiotic resistance profiles of 50 clinical isolates of *P. aeruginosa* against ciprofloxacin, gentamicin, and imipenem are presented in Figure 3. In the figure, color coding is used to indicate resistance patterns: red denotes resistance, green indicates susceptibility, and yellow represents intermediate susceptibility.

Figure 3Antibiotic resistance profiles of 50 clinical isolates of *P. aeruginosa* against ciprofloxacin, gentamicin, and imipenem (red: resistance; green: sensitive; yellow: intermediate).

**Figure:**
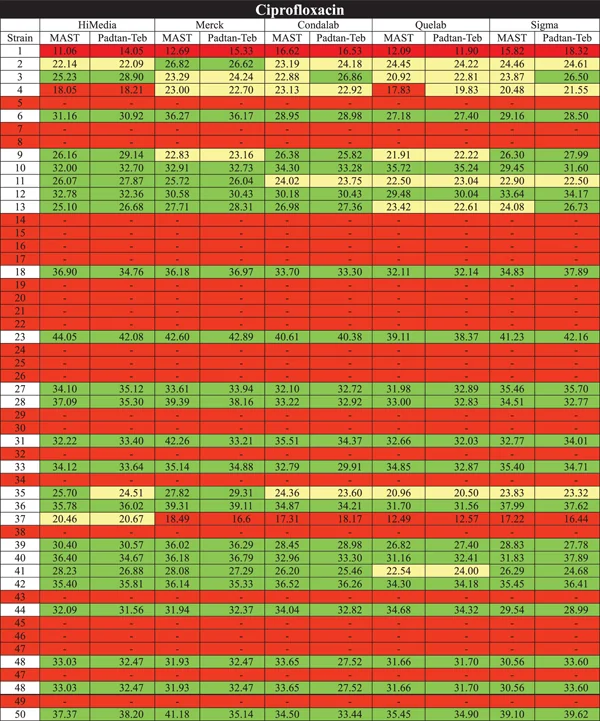

**Figure:**
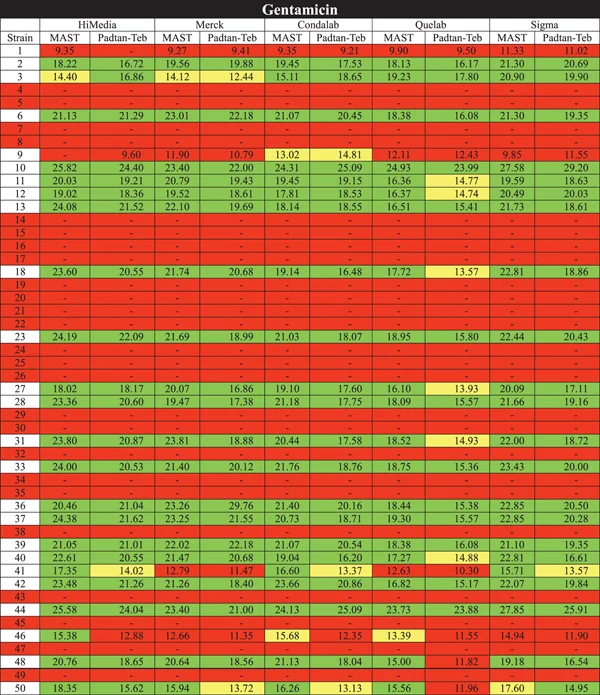

**Figure:**
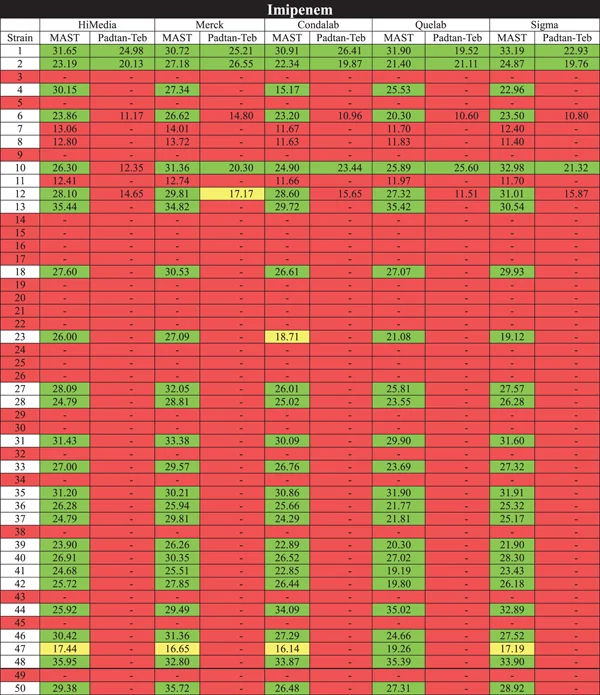

Figure 3 indicates that the choice of commercial MHA affected not only inhibition‐zone diameters but also the categorical interpretation of susceptibility among clinical *P. aeruginosa* isolates. Media such as Quelab and Condalab, which generally produced smaller inhibition zones, shifted some isolates toward more resistant interpretations, whereas Sigma or Merck more often yielded larger zones and more susceptible classifications depending on the antibiotic tested. These findings show that differences among MHA brands can modify the reported susceptibility profile of the same isolate and therefore influence the clinical interpretation of antibiogram data. Table 5 summarizes the antibiotic‐level pattern of susceptibility, zone‐direction shifts by medium, and the strongest clinically relevant disagreement signals obtained from Figure 3.

**Table 5:** Antibiotic‐level pattern of 50 *P. aeruginosa* isolates, zone‐direction shifts by medium, and the strongest clinically relevant disagreement signals obtained from Figure 3.

Antibiotic	Medium with largest zones/highest sensitivity	Medium with smallest zones/lowest sensitivity	Clinical interpretation impact
**Gentamicin**	**Sigma**	**Quelab**	**Borderline isolates would shift toward R/I on Quelab medium and toward S on Sigma medium**
**Imipenem**	**Merck**	**Quelab**	**Strongest instability of imipenem disks; 23 susceptible isolates were only tested with Mast disks, not both brands**
**Ciprofloxacin**	**—**	**Quelab and Condalab**	**Category shifts are likely for isolates near CLSI breakpoints because significantly different zones were observed**

As shown in Figure 3, 23 clinical strains of *P. aeruginosa* were susceptible to ciprofloxacin, whereas the remaining strains exhibited resistance pattern. Among the ciprofloxacin‐susceptible isolates, the highest levels of sensitivity, as indicated by larger inhibition zone diameters, were observed on HiMedia and Merck culture media, whereas the lowest sensitivity was recorded on Quelab medium (Figure 4A).

**Figure 4:**
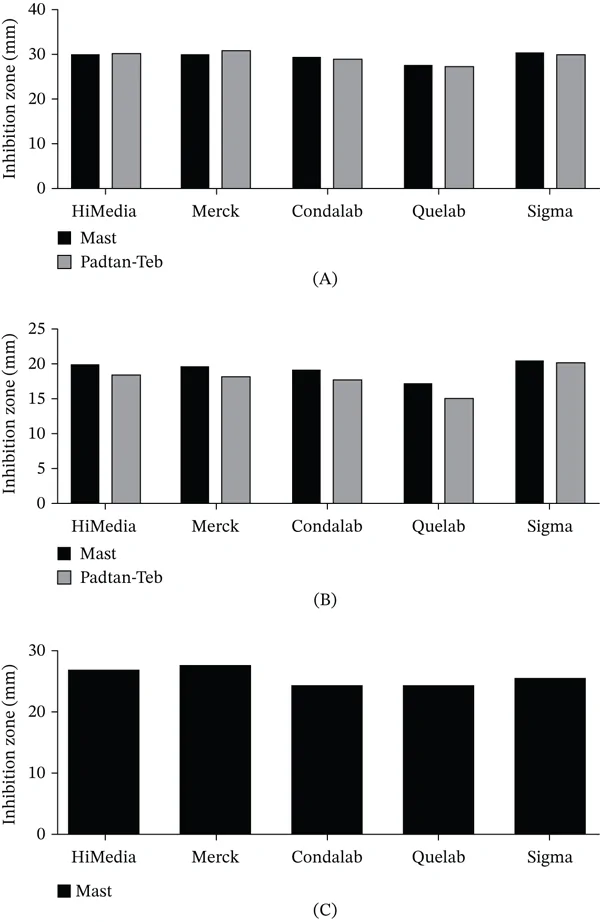
Comparative chart of inhibition zone diameters (mm) for clinical *P. aeruginosa* strains exposed to (A) ciprofloxacin, (B) gentamicin, and (C) imipenem.

Similarly, 22 strains demonstrated susceptibility to gentamicin, with the remaining isolates showing resistance. For this antibiotic, the largest inhibition zones were observed on Sigma medium, whereas the smallest were again recorded on Quelab medium (Figure 4B).

According to the data presented, only three strains were susceptible to both imipenem disks (from different manufacturers), whereas 23 strains exhibited susceptibility exclusively to the disks produced by Mast. Due to the lack of measurable response with the imipenem disks from Padtan‐Teb, the comparative analysis of average inhibition zones was conducted using only the Mast disks. In this analysis, Merck medium yielded the highest sensitivity to imipenem, whereas Quelab medium showed the lowest (Figure 4C).

The appearance of one of the clinical strains of *P. aeruginosa* on these five MHA media was also examined (Figure 5). A range of colors including pale or deep yellow, green, and blue was seen in different brands of MHA medium, which may be due to the production of different pigments in relation to the variable amount of cations present in the agar medium.

**Figure 5:**
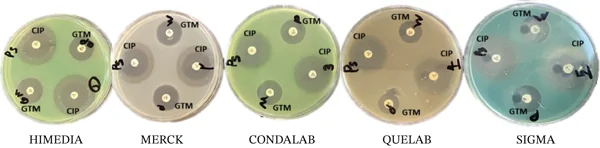
Color variations observed among five commercial MHA media used for performing antibiogram tests on one of the clinical *P. aeruginosa* isolates, highlighting differences in the composition of each medium.

## 4. Discussion

The present findings indicate that commercial MHA formulation and inoculation methodology can contribute to variability in disk diffusion testing of *P. aeruginosa*. The following sections consider the potential contribution of medium composition, agar microstructure, and inoculation methodology to the observed variability.

Precise assessment of antibiotic efficacy and bacterial susceptibility is essential in clinical microbiology and for selecting appropriate antimicrobial treatments. One important, but frequently neglected, variable affecting susceptibility test outcomes, especially for specific antibiotic groups, is the cation concentration in MHA [14]. This parameter can markedly influence the activity of antibiotics such as ciprofloxacin, imipenem, and gentamicin, and therefore warrants careful attention when discussing the accuracy of susceptibility testing in scientific manuscripts.

In the case of aminoglycoside antibiotics such as gentamicin, elevated levels of divalent cations in MHA can diminish their apparent activity against certain bacterial species, particularly *P. aeruginosa*. This inhibitory effect arises because divalent cations compete with the positively charged aminoglycosides for binding sites on the bacterial outer membrane, thereby restricting antibiotic uptake into the cell. Conversely, when cation concentrations are low, aminoglycoside activity may appear artificially enhanced. Studies have shown that the zinc content of MHA, along with other cations, strongly correlates with the MICs of gentamicin, tobramycin, and amikacin against *P. aeruginosa*. These findings underscore the importance of maintaining standardized cation concentrations in MHA to achieve consistent and clinically meaningful susceptibility results for aminoglycosides [25]. For example, Lee et al. demonstrated how varying gentamicin concentrations influence bacterial populations over time, emphasizing the antibiotic′s sensitivity to environmental conditions [26].

Ciprofloxacin, a member of the fluoroquinolone class, exhibits variations in activity depending on the cation concentration present in the testing medium. Divalent cations can chelate fluoroquinolones, forming complexes that hinder the antibiotic′s penetration into bacterial cells and its interaction with intracellular targets such as DNA gyrase and topoisomerase IV [27]. This chelation effect may result in artificially elevated MICs and consequently an overestimation of resistance when cation levels in MHA are high. Differences in cation concentrations have been suggested as a contributing factor to the wide range of ciprofloxacin susceptibility results reported across studies. For instance, resistance to ciprofloxacin varies markedly among bacterial isolates, from nearly 0% in community‐associated MRSA (CA‐MRSA) to approximately 60%–70% in hospital‐associated MRSA (HA‐MRSA) [28]. Similarly, *E. coli* isolates from wild black grouse were found to be fully susceptible to ciprofloxacin in one study, whereas swine‐derived *E. coli* isolates demonstrated 43.5% resistance and 56.5% susceptibility [29, 30]. Such variability highlights the critical importance of maintaining consistent cation concentrations during testing.

Imipenem, a carbapenem‐class antibiotic, is generally less influenced by cation concentration than aminoglycosides or fluoroquinolones; however, its activity is not completely unaffected by these factors. Elevated cation levels may occasionally lead to a slight reduction in apparent activity, particularly among isolates with borderline susceptibility.

It should be noted that the elemental concentrations measured in the present study should not be interpreted against predefined CLSI or EUCAST acceptance ranges for MHA. Quality control of MHA for AST is primarily performance‐based, whereby the prepared medium is evaluated using appropriate reference strains to verify that the resulting inhibition‐zone diameters fall within established acceptable ranges for the tested antimicrobial agents. Therefore, differences in Ca, Mg, Zn, and other elemental concentrations among the commercial MHA brands observed in this study represent differences in their chemical composition rather than, by themselves, evidence of failure to meet international AST quality‐control requirements.

The present findings are in agreement with previous evaluations indicating that commercially available MHAs cannot be considered analytically interchangeable. In an EUCAST evaluation of 21 dehydrated MH media, only six brands demonstrated excellent overall performance, whereas several others showed considerable frequencies of out‐of‐range quality‐control results, with discrepancies occurring particularly frequently for aminoglycosides and fluoroquinolones [31]. Similarly, a subsequent EUCAST investigation of 10 brands of prepoured MHA plates identified significant differences in performance among manufacturers; notably, HiMedia plates were among the poorer‐performing products [13]. This observation is particularly relevant to the present study, which also included HiMedia MHA as one of the evaluated brands. More recent investigations involving cefiderocol have further supported this general observation, demonstrating that the choice of MHA brand may produce shifts in inhibition‐zone diameters of sufficient magnitude to influence susceptibility interpretation, including among *P. aeruginosa* isolates tested on different commercial media [32, 33].

Differences in cation concentrations among various commercial brands of MHA have been directly examined. One study comparing four different MHA brands found notable discrepancies in polymyxin B susceptibility results, which were attributed to variations in their cation content [14]. Although this investigation specifically addressed polymyxin B, the underlying principle extends to other antibiotics whose activity is influenced by cation levels. Similarly, another study assessing disk diffusion testing for cefiderocol across different MHA brands highlighted the lack of standardization and its potential impact on the reliability of susceptibility testing outcomes [18].

An additional consideration is the marked difference observed between the imipenem disks from the two manufacturers. Although the disks were within their stated expiration dates and were stored under appropriate conditions, the Padtan‐Teb imipenem disks showed substantially poorer performance than the Mast disks. This finding may reflect differences in product potency, stability, or manufacturing quality. This possibility is particularly relevant for imipenem, which is a relatively unstable antimicrobial and may be susceptible to loss of activity if appropriate manufacturing, formulation, packaging, and quality‐control measures are not adequately maintained. However, because the present study did not include a quantitative potency assay or a dedicated stability assessment of the antibiotic disks, a specific manufacturing defect or degradation mechanism cannot be established. Therefore, this observation should be considered a potential product‐quality issue that warrants further investigation using potency testing and stability studies across multiple production lots.

Furthermore, the differences summarized in Figure 3 have direct clinical importance because they show that variation among commercial MHA brands can change the categorical interpretation of susceptibility for the same *P. aeruginosa* isolate. Media that produced smaller inhibition zones, particularly Quelab and Condalab, would be expected to classify borderline isolates as more resistant, whereas media producing larger zones could shift interpretation toward susceptibility. Thus, these inconsistencies can compromise the clinical interpretation of antibiogram data by introducing medium‐dependent differences into patient‐facing susceptibility reports. Given that calcium and magnesium concentrations varied markedly among the tested media, the observed discrepancies likely reflect compositional effects on antibiotic activity, although the present data establish association rather than direct causation.

The observed association between reduced inhibition‐zone diameters and higher cation concentrations is consistent with earlier mechanistic investigations involving *P. aeruginosa*. Previous studies demonstrated an inverse relationship between gentamicin zone diameters and both ionized calcium and the combined soluble calcium and magnesium concentrations in MHA, suggesting that relatively small differences in the physicochemical composition of the medium may have a substantial influence on apparent antimicrobial susceptibility [15, 34]. Experimental addition of calcium and magnesium to cation‐deficient media resulted in smaller inhibition zones and produced performance characteristics comparable to those of media with higher native cation concentrations, providing further evidence for the contribution of these ions to the observed differences [15, 34]. The potential clinical significance of this effect is underscored by reports that elevated magnesium or calcium concentrations can increase aminoglycoside MICs by 16 to 32‐fold. Likewise, supplementation with physiological concentrations of free calcium and magnesium has been shown to substantially increase gentamicin MICs among clinical *P. aeruginosa* isolates, with reported resistance rates increasing from 2% to 78% under supplemented conditions [35].

The impact of gel microstructure, particularly the presence and dimensions of capillary spaces, is an important consideration in understanding molecular transport in gel systems. In the present study, microscopic observations revealed only slight variations in the gel structure among the tested media. The observed microstructures appeared to provide pathways compatible with antibiotic diffusion under the conditions examined, considering the molecular sizes of the tested antibiotics. However, these observations should be interpreted cautiously, as diffusion coefficients and diffusion kinetics were not directly measured in the present study. Diffusion within gel systems is a multifaceted process influenced by several factors, including the size and shape of the diffusing molecules, concentration gradients, and the physical characteristics of the gel matrix, such as pore size, tortuosity, and the presence of interconnected capillary spaces. Gel structures are often heterogeneous, consisting of interlinked polymer networks that form a porous environment through which solutes must traverse. Although the movement of substances through such systems is generally described by Fick′s laws of diffusion, quantitative assessment of diffusion in a gel matrix typically requires consideration of the restrictive effects imposed by its structural characteristics. Therefore, the minor differences observed in gel microstructure in the present study do not, by themselves, establish the absence or presence of a significant effect on antibiotic diffusion. [36–38].

The observed differences in colony pigmentation among *P. aeruginosa* isolates grown on the commercial MHA media represent an additional phenotypic manifestation of differences in the growth environment. *P. aeruginosa* produces several pigments, including pyocyanin, pyoverdine, pyorubin, and pyomelanin, whose expression can vary depending on bacterial genotype and environmental and nutritional conditions [39–43]. In particular, pyoverdine production is regulated by iron availability, with increased production generally occurring under iron‐limited conditions [44]. Other pigments, such as pyocyanin, are also regulated by complex physiological and quorum‐sensing mechanisms [43, 45–47]. Therefore, differences in the chemical environment of MHA, including the availability of metal ions such as iron, may influence pigment expression and other physiological characteristics of *P. aeruginosa*. However, in the present study, pigment production was assessed qualitatively and was not quantitatively compared with inhibition‐zone diameters or susceptibility classifications. Thus, the observed pigmentation differences cannot be considered a demonstrated confounding factor for the AST results. Nevertheless, because variations in the chemical environment of the medium may affect both bacterial physiology and antimicrobial activity, an indirect relationship between medium composition, pigment expression, and AST performance cannot be excluded and warrants further investigation. Although colony pigmentation may provide a useful phenotypic characteristic for preliminary characterization of *P. aeruginosa* [48, 49], its specific relevance to AST requires dedicated experimental evaluation.

The double‐layer inoculation method for AST produces lower variability and more consistent inhibition zones than the standard swab method, indicating superior reproducibility. This improved uniformity arises from a more evenly distributed bacterial lawn, which ensures that antibiotic diffusion interacts with a consistent cell density, reducing discrepancies in inhibition zone measurements. Enhanced reproducibility increases confidence in susceptibility results, particularly near breakpoint values, decreases the need for repeat testing, and supports inter‐laboratory comparability, all of which are critical for clinical decision‐making and antimicrobial resistance surveillance. However, adopting the double‐layer method involves trade‐offs, including increased procedural complexity, time, labor, material costs, and the need for staff training and validation, compared with the simpler, faster, and cost‐effective CLSI swab method. Despite these challenges, the double‐layer approach may be justified for antibiotic‐bacterium combinations prone to high variability or in research settings where precision is paramount, balancing improved data quality against practical laboratory considerations to ensure accurate and reliable AST outcomes [13, 50].

From a practical perspective, although the double‐layer technique demonstrated lower variability in the present study, its relatively laborious and time‐consuming procedure may limit its application in high‐throughput routine clinical microbiology laboratories. It may be more suitable for research settings or laboratories with a relatively low workload where improved methodological reproducibility and accuracy are prioritized. In the present study, the double‐layer technique was therefore selected primarily as a research‐oriented approach to minimize variability associated with inoculation and to enable a more reliable comparison of different commercial MHA formulations. Thus, our findings should not be interpreted as suggesting that the double‐layer method should replace conventional swabbing in routine clinical AST, but rather that it may serve as a useful approach when experimental control and reproducibility are particularly important.

The finding that the double‐layer inoculation technique yielded the lowest variability should be considered alongside broader evidence demonstrating the sensitivity of disk diffusion testing to operator‐dependent procedures. Previous standardization studies have shown that factors such as inoculum density, plate streaking, and inhibition‐zone measurement can substantially affect the precision and accuracy of disk diffusion results, whereas automated inoculation has been reported to reduce both interoperator and overall methodological variability compared with manual procedures [51]. Evaluations conducted in routine laboratory settings have likewise identified inoculum preparation, manual streaking, disk placement, incubation conditions, and subjective zone measurement as important sources of variation affecting test repeatability [52]. Within this context, the present results suggest that differences related to the culture medium may interact with, or be influenced by, the inoculation procedure, potentially amplifying or reducing the variability observed during AST. Thus, optimization and standardization of the plating technique may represent an additional strategy for improving reproducibility, particularly in laboratories relying on manual disk diffusion workflows [52].

Despite the comprehensive analysis presented in this study, several limitations should be acknowledged. First, the work focused exclusively on *P. aeruginosa* and three antibiotics, imipenem, ciprofloxacin, and gentamicin, limiting the generalizability of the findings to other bacterial species and antibiotic classes. Second, although five commercial MHA brands were compared, batch‐to‐batch variability within each brand was not assessed, which may further influence susceptibility test results in routine laboratory settings. Third, although ICP‐MS analysis demonstrated considerable variation in divalent cation concentrations, particularly calcium and magnesium, among the commercial MHA brands, these measurements were obtained from commercially formulated media rather than from a controlled series with systematically varied cation concentrations. Therefore, a concentration‐response or causal relationship between individual cations and inhibition‐zone diameter cannot be established from the present data, and the observed findings should be interpreted as an association between differences in elemental composition and variability in AST results. Other medium components and environmental factors, such as pH fluctuations or organic impurities, were also not investigated and may contribute to antibiotic activity. Moreover, microscopic analysis revealed only minor differences in gel microstructure, and more advanced structural characterization may be required to fully elucidate their potential effects on antibiotic diffusion. Future studies should therefore include a broader range of bacterial species and antibiotics, assess longitudinal batch‐to‐batch variability, and employ controlled MHA formulations with systematically varied cation concentrations to further clarify the factors contributing to variability in AST results.

These findings have important implications for routine clinical microbiology practice. EUCAST methodological guidance recommends that each new batch of MHA be locally verified against established quality‐control limits for the antimicrobial agents routinely tested, given that both inter‐manufacturer and intra‐manufacturer batch variability may influence inhibition‐zone diameters [53]. This recommendation is particularly relevant in light of surveys indicating that internal quality control is not consistently implemented across laboratories and that nonstandard media may sometimes be used. Such practices have been associated with compromised AST performance and increased error rates, as demonstrated when nutrient agar was used in place of MHA [54]. Accordingly, the relevance of the present findings extends beyond experimental settings to routine patient care, since media‐associated shifts in inhibition‐zone diameters may have greater consequences for isolates with measurements close to interpretive breakpoints or for antimicrobial agents whose susceptibility results are particularly sensitive to medium composition, including aminoglycosides, fluoroquinolones, polymyxins, and cefiderocol.

## 5. Conclusion

This study demonstrates that the performance of *P. aeruginosa* AST by the disk diffusion method varies significantly among commercial MHA brands. Although all tested commercial MHAs adequately supported bacterial growth, significant differences in inhibition‐zone diameters and susceptibility profiles were observed across the media. In particular, Quelab and Condalab generally produced smaller inhibition zones for several of the tested antibiotics, whereas the magnitude of inhibition varied according to the antibiotic and MHA brand. ICP‐MS analysis demonstrated substantial differences in the elemental composition of the tested media, particularly in calcium, magnesium, and zinc concentrations, which were observed alongside variations in antimicrobial susceptibility results. However, these findings indicate an association rather than a demonstrated causal effect of individual cations on inhibition‐zone diameters. Microscopic examination revealed only minor differences in agar gel microstructure, with no major differences in antibiotic diffusion patterns observed. Among the evaluated inoculation techniques, the double‐layer method produced the lowest coefficient of variation and therefore showed the highest reproducibility under the conditions evaluated in this study. However, this finding should not be interpreted as evidence of superior accuracy or overall clinical performance, as accuracy was not independently assessed against CLSI or EUCAST reference performance criteria, and interoperator variability was not evaluated. Overall, the main findings of this study are that commercial MHA brands can yield different disk diffusion results for *P. aeruginosa*, that substantial differences in medium elemental composition accompany this variability, and that the double‐layer technique provides greater reproducibility than the swab and pour‐plate methods under the experimental conditions examined. These findings support the importance of appropriate quality control and standardization of commercial MHA and careful consideration of inoculation methodology when reproducible AST results are required.

## Author Contributions

Nasrin Samadi and Amir Reza Abdollahi: conceptualization, project administration, supervision. Seyed Sadeq Mousavi Ghahfarrokhi: writing, review, and editing. Fatemeh Aghajafari, Seyed Sadeq Mousavi Ghahfarrokhi, Hossein Jafari‐Nodoushan, and Marjan Tootianquds: methodology, validation, and formal analysis. Amir Reza Abdollahi and Seyed Sadeq Mousavi Ghahfarrokhi have contributed to the work equally and should be regarded as co‐first authors.

## Funding

This study was supported by the Tehran University of Medical Sciences and Health Services (10.13039/501100004484; 67167) and the National Institute for Medical Research Development (NIMAD) (4002378).

## Ethics Statement

This study was approved by the Ethics Committee of Tehran University of Medical Sciences (Ethics Approval No. IR.TUMS.BLC.1402.024). Clinical bacterial isolates were obtained from specimens collected from patients at the participating hospitals as part of routine clinical diagnostic procedures. The isolates were subsequently used for laboratory‐based microbiological investigations. All clinical isolates and associated information were deidentified prior to their inclusion in the study, and no patient‐identifying information was accessed, recorded, or reported. In accordance with the approval of the institutional Ethics Committee and the anonymized nature of the study, informed consent from individual patients was not required.

## Conflicts of Interest

The authors declare no conflicts of interest.

## Data Availability

Data available on request from the authors.

## References

[1] Qin S. , Xiao W. , Zhou C. , Pu Q. , Deng X. , Lan L. , Liang H. , Song X. , and Wu M. , *Pseudomonas aeruginosa*: Pathogenesis, Virulence Factors, Antibiotic Resistance, Interaction With Host, Technology Advances and Emerging Therapeutics, Signal Transduction and Targeted Therapy. (2022) 7, no. 1, 10.1038/s41392-022-01056-1, 35752612. PMC9233671 35752612

[2] Diggle S. P. and Whiteley M. , Microbe Profile: *Pseudomonas aeruginosa*: Opportunistic Pathogen and Lab Rat, Microbiology. (2020) 166, no. 1, 30–33, 10.1099/mic.0.000860. PMC7273324 31597590

[3] Elfadadny A. , Ragab R. F. , AlHarbi M. , Badshah F. , Ibáñez-Arancibia E. , Farag A. , Hendawy A. O. , de los Ríos-Escalante P. R. , Aboubakr M. , Zakai S. A. , and Nageeb W. M. , Antimicrobial Resistance of *Pseudomonas aeruginosa*: Navigating Clinical Impacts, Current Resistance Trends, and Innovations in Breaking Therapies, Frontiers in Microbiology. (2024) 15, 1374466, 10.3389/fmicb.2024.1374466, 38646632. PMC11026690 38646632

[4] Valero A. , Isla A. , Rodríguez-Gascón A. , Canut A. , and Ángeles Solinís M. , Susceptibility of *Pseudomonas aeruginosa* and Antimicrobial Activity Using PK/PD Analysis: an 18-Year Surveillance Study, Enfermedades Infecciosas y Microbiologia Clinica. (2019) 37, no. 10, 626–633, 10.1016/j.eimc.2019.02.009, 31005313. 31005313

[5] Murata M. , Kosai K. , Mitsumoto-Kaseida F. , Kaku N. , Hasegawa H. , Izumikawa K. , Mukae H. , and Yanagihara K. , Antimicrobial Susceptibility and Resistance Mechanisms to Antipseudomonal *β*-Lactams in *Pseudomonas aeruginosa* Isolates From Blood, Microbiology Spectrum. (2025) 13, no. 5, e0279024, 10.1128/spectrum.02790-24, 40162762. PMC12054155 40162762

[6] Pathmanathan S. G. , Samat N. A. , and Mohamed R. , Antimicrobial Susceptibility of Clinical Isolates of *Pseudomonas aeruginosa* From a Malaysian Hospital, Malaysian journal of medical sciences: MJMS. (2009) 16, no. 2, 27–32, 22589655. PMC3336164 22589655

[7] Buyukyanbolu E. , Genc L. , Cyr E. A. , Karakus M. , Comert F. , Otlu B. , Aktas E. , and Nicolau D. P. , Antimicrobial Susceptibility Profile of Ceftolozane/Tazobactam, Ceftazidime/Avibactam and Cefiderocol Against Carbapenem-Resistant *Pseudomonas aeruginosa* Clinical Isolates From Türkiye, European Journal of Clinical Microbiology and Infectious Diseases. (2024) 43, no. 9, 1787–1794, 10.1007/s10096-024-04896-7, 38995343. 38995343

[8] Perez F. , El Chakhtoura N. G. , and Bonomo R. A. , Management of Severe Infections: Multidrug-Resistant and Carbapenem-Resistant Gram-Negative Bacteria, Medical Clinics of North America. (2025) 109, no. 3, 735–747, 10.1016/j.mcna.2025.01.003. 40185559

[9] Fouad A. , Nicolau S. E. , Tamma P. D. , Simner P. J. , Nicolau D. P. , and Gill C. M. , Differential Frequency of Persister Cells in Clinically Derived Isolates of *Pseudomonas aeruginosa* After Exposure to Cefiderocol and Ceftolozane/Tazobactam, Journal of Antimicrobial Chemotherapy. (2024) 79, no. 12, 3236–3242, 10.1093/jac/dkae346, 39442926. 39442926

[10] Murray P. R. and Zeitinger J. R. , Evaluation of Mueller-Hinton Agar for Disk Diffusion Susceptibility Tests, Journal of Clinical Microbiology. (1983) 18, no. 5, 1269–1271, 10.1128/jcm.18.5.1269-1271.1983, 6643674. PMC272882 6643674

[11] Leber A. L. , Disk Diffusion Test, Clinical Microbiology Procedures Handbook, 2016, ASM Press, 10.1128/9781555818814.ch5.1.

[12] Institute, Clinical and Laboratory Standards , Performance Standards for Antimicrobial Susceptibility Testing, 2023, 33rd edition, CLSI Supplement M100.

[13] Åhman J. , Matuschek E. , and Kahlmeter G. , Evaluation of Ten Brands of Pre-Poured Mueller-Hinton Agar Plates for EUCAST Disc Diffusion Testing, Clinical Microbiology and Infection. (2022) 28, no. 11, 1499.e1–1499.e5, 10.1016/j.cmi.2022.05.030, 35659925. 35659925

[14] Girardello R. , Bispo P. J. M. , Yamanaka T. M. , and Gales A. C. , Cation Concentration Variability of Four Distinct Mueller-Hinton Agar Brands Influences Polymyxin B Susceptibility Results, Journal of Clinical Microbiology. (2012) 50, no. 7, 2414–2418, 10.1128/JCM.06686-11, 22553247. PMC3405622 22553247

[15] Casillas E. , Kenny M. A. , Minshew B. H. , and Schoenknecht F. D. , Effect of Ionized Calcium and Soluble Magnesium on the Predictability of the Performance of Mueller-Hinton Agar Susceptibility Testing of *Pseudomonas aeruginosa* With Gentamicin, Antimicrobial Agents and Chemotherapy. (1981) 19, no. 6, 987–992, 10.1128/AAC.19.6.987, 6791589. PMC181596 6791589

[16] Wang S. , McDonnell E. H. , Sedor F. A. , and Toffaletti J. G. , pH Effects on Measurements of Ionized Calcium and Ionized Magnesium in Blood, Archives of Pathology & Laboratory Medicine. (2002) 126, no. 8, 947–950, 10.5858/2002-126-0947-PEOMOI. 12171493

[17] Høiby N. , Moser C. , and Ciofu O. , The Microenvironment in Antibiotic Susceptibility Testing, APMIS: Acta Pathologica, Microbiologica, et Immunologica Scandinavica. (2024) 132, no. 12, 985–991, 10.1111/apm.13405. 38565324

[18] Potter R. F. , Wallace M. A. , Muenks C. E. , Alvarado K. , Yarbrough M. L. , and Burnham C. A. D. , Evaluation of Variability in Interpretation of Disk Diffusion Testing for Cefiderocol Using Different Brands of Mueller-Hinton Agar, Journal of Applied Laboratory Medicine. (2023) 8, no. 3, 523–534, 10.1093/jalm/jfac131, 36738243. 36738243

[19] D′Amato R. F. and Thornsberry C. , Calcium and Magnesium in Mueller-Hinton Agar and Their Influence on Disk Diffusion Susceptibility Results, Current Microbiology. (1979) 2, no. 3, 135–138, 10.1007/BF02605869.

[20] Olaru I. D. , Schoeler S. , and Schaumburg F. , The Impact of Agar Depth on Antimicrobial Susceptibility Testing by Disc Diffusion, Journal of Medical Microbiology. (2024) 73, no. 9, 10.1099/jmm.0.001890, 39292220. PMC11410041 39292220

[21] Feleke M. G. , Edao M. H. , Yilhal G. A. , Mekasha Y. T. , Mekonen A. W. , Fenta M. D. , Mengistu B. A. , Berihun A. M. , Getnet M. A. , Demessie Y. , and Getnet K. , Isolation and Identification of *Pseudomonas fluoeroscens* and Evaluation of Its Antimicrobial Sensitivity From Fish in Lake Haramaya, East Hararghe Zone, Oromia Regional State, Ethiopia, Scientific Reports, 2026, Nature Publishing Group, 10.1038/s41598-026-65235-8.

[22] Balouiri M. , Sadiki M. , and Ibnsouda S. K. , Methods for In Vitro Evaluating Antimicrobial Activity: A Review, Journal of Pharmaceutical Analysis. (2016) 6, no. 2, 71–79, 10.1016/j.jpha.2015.11.005, 29403965. PMC5762448 29403965

[23] CLSI , Attachment D CRE Instrument Attachment Laboratory Guidelines, 2016, CLSI.

[24] Erickson H. P. , Size and Shape of Protein Molecules at the Nanometer Level Determined by Sedimentation, Gel Filtration, and Electron Microscopy, Biological Procedures Online. (2009) 11, no. 1, 32–51, 10.1007/s12575-009-9008-x, 19495910. PMC3055910 19495910

[25] Washington J. A. , Snyder R. J. , Kohner P. C. , Wiltse C. G. , Ilstrup D. M. , and McCall J. T. , Effect of Cation Content of Agar on the Activity of Gentamicin, Tobramycin, and Amikacin Against *Pseudomonas aeruginosa* , Journal of Infectious Diseases. (1978) 137, no. 2, 103–111, 10.1093/infdis/137.2.103, 415096. 415096

[26] Lee K.-S. , Lee S.-M. , Oh J. , Park I. H. , Song J. H. , Han M. , Yong D. , Lim K. J. , Shin J. S. , and Yoo K. H. , Electrical Antimicrobial Susceptibility Testing Based on Aptamer-Functionalized Capacitance Sensor Array for Clinical Isolates, Scientific Reports. (2020) 10, no. 1, 13709, 10.1038/s41598-020-70459-3, 32792573. PMC7426404 32792573

[27] Bloise I. , Guedez-López G. V. , Tejedor-Rodríguez M. , Romero-Gómez M. P. , García-Rodríguez J. , Mingorance J. , and Cendejas-Bueno E. , Bloodstream Infection due to *Herbaspirillum* sp. Case Series and Review of Literature, European Journal of Clinical Microbiology and Infectious Diseases. (2021) 40, no. 4, 779–785, 10.1007/s10096-020-04075-4, 33083918. 33083918

[28] Sohail M. , Muzzammil M. , Ahmad M. , Rehman S. , Garout M. , Khojah T. M. , al-Eisa K. M. , Breagesh S. A. , Hamdan R. M. A. , Alibrahim H. I. , Alsoliabi Z. A. , Rabaan A. A. , and Ahmed N. , Molecular Characterization of Community- and Hospital-Acquired Methicillin-Resistant *Staphylococcus aureus* Isolates During COVID-19 Pandemic, Antibiotics. (2023) 12, no. 1, 10.3390/antibiotics12010157, 36671358. PMC9854722 36671358

[29] Silva A. , Silva V. , Dapkevicius M. d. L. E. , Dapkevicius M. L. E. , Azevedo M. , Cordeiro R. , Pereira J. E. , Valentão P. , Falco V. , Igrejas G. , Caniça M. , and Poeta P. , Unveiling Antibiotic Resistance, Clonal Diversity, and Biofilm Formation in E. coli Isolated From Healthy Swine in Portugal, Pathogens. (2024) 13, no. 4, 10.3390/pathogens13040305, 38668260. PMC11054063 38668260

[30] Kwaśna N. , Majewska M. , Karwańska M. , Siedlecka M. , Pałucki A. , and Piasecki T. , Occurrence and Molecular Characterization of *Escherichia coli* Strains Isolated From Black Grouse (*Lyrurus tetrix*) From the Karkonosze National Park in Poland, BMC Veterinary Research. (2024) 20, no. 1, 10.1186/s12917-024-03886-3, 38297324. PMC10829180 38297324

[31] Åhman J. , Matuschek E. , and Kahlmeter G. , EUCAST Evaluation of 21 Brands of Mueller-Hinton Dehydrated Media for Disc Diffusion Testing, Clinical Microbiology and Infection. (2020) 26, no. 10, 1412.e1–1412.e5, 10.1016/j.cmi.2020.01.018, 32006695. 32006695

[32] Potter R. F. , Wallace M. A. , Muenks C. E. , Alvarado K. , Yarbrough M. L. , and Burnham C. A. D. , Evaluation of Variability in Interpretation of Disk Diffusion Testing for Cefiderocol Using Different Brands of Mueller–Hinton Agar, Journal of Applied Laboratory Medicine. (2023) 8, no. 3, 523–534, 10.1093/jalm/jfac131, 36738243. 36738243

[33] Saar M. , Wawrzyk A. , Pastuszak-Lewandoska D. , and Bielec F. , Cefiderocol Antimicrobial Susceptibility Testing by Disk Diffusion: Influence of Agar Media and Inhibition Zone Morphology in *K. pneumoniae* Metallo-*β*-Lactamase, Antibiotics. (2025) 14, no. 5, 10.3390/antibiotics14050527, 40426593. PMC12108340 40426593

[34] Kenny M. A. , Pollock H. M. , Minshew B. H. , Casillas E. , and Schoenknecht F. D. , Cation Components of Mueller-Hinton Agar Affecting Testing of *Pseudomonas aeruginosa* Susceptibility to Gentamicin, Antimicrobial Agents and Chemotherapy. (1980) 17, no. 1, 55–62, 10.1128/AAC.17.1.55, 6766293. PMC283726 6766293

[35] Reller L. B. , Schoenknecht F. D. , Kenny M. A. , and Sherris J. C. , Antibiotic Susceptibility Testing of *Pseudomonas aeruginosa*: Selection of a Control Strain and Criteria for Magnesium and Calcium Content in Media, Journal of infectious diseases. (1974) 130, no. 5, 454–463, 10.1093/infdis/130.5.454, 4370727. 4370727

[36] Muhr A. H. and Blanshard J. M. , Diffusion in Gels, Polymer. (1982) 23, no. 7, 1012–1026, 10.1016/0032-3861(82)90402-5.

[37] Buck K. K. S. , Dungan S. R. , and Phillips R. J. , The Effect of Solute Concentration on Hindered Gradient Diffusion in Polymeric Gels, Journal of Fluid Mechanics. (1999) 396, 287–317, 10.1017/S0022112099006035.

[38] Kojic M. , Milosevic M. , Kojic N. , Koay E. J. , Fleming J. B. , Ferrari M. , and Ziemys A. , Mass Release Curves as the Constitutive Curves for Modeling Diffusive Transport Within Biological Tissue, Computers in Biology and Medicine. (2018) 92, 156–167, 10.1016/j.compbiomed.2016.06.026, 29182964. PMC5927384 29182964

[39] Kothari A. , Yadav R. , Rajput P. , Singh V. , Kaushal K. , Jain N. , and Omar B. J. , Multidrug Resistance Behaviors of Clinical Pseudomonas aeruginosa Strains Associated to Pigment Coloration, 2021, Preprints.org, 10.20944/preprints202106.0192.v1.

[40] Kothari A. , Kumar S. K. , Singh V. , Kumar P. , Kaushal K. , Pandey A. , Jain N. , and Omar B. J. , Association of Multidrug Resistance Behavior of Clinical *Pseudomonas aeruginosa* to Pigment Coloration, European Journal of Medical Research. (2022) 27, no. 1, 10.1186/s40001-022-00752-6, 35842687. PMC9288039 35842687

[41] Orlandi V. T. , Martegani E. , Giaroni C. , Baj A. , and Bolognese F. , Bacterial Pigments: A Colorful Palette Reservoir for Biotechnological Applications, Biotechnology and Applied Biochemistry. (2022) 69, no. 3, 981–1001, 10.1002/bab.2170, 33870552. PMC9544673 33870552

[42] Nedeljković N. S. , Kocić B. , Tiodorović B. , Branković S. , and Antić S. M. , Serotyping and Analysis of Produced Pigments Kinds by *Pseudomonas aeruginosa* Clinical Isolates, Vojnosanitetski Pregled. (2011) 68, no. 11, 923–929, 10.2298/VSP1111923S, 22191308. 22191308

[43] Nakata M. , Studies on the Pigment Production of *Pseudomonas aeruginosa* , Journal of Kansai Medical University. (1963) 15, no. 1, 37–42, 10.5361/jkmu1956.15.1_37.

[44] van Genechten W. , van Dijck P. , and Demuyser L. , Fluorescent Toys ′n′ Tools Lighting the Way in Fungal Research, FEMS Microbiology Reviews. (2021) 45, no. 5, 15746976, 10.1093/femsre/fuab013. PMC8498796 33595628

[45] Li P. , Pan J. , Dong Y. , Sun Y. , Wang Y. , Liao K. , Chen Y. , Deng X. , Yu S. , and Hu H. , Microenvironment Responsive Charge-Switchable Nanoparticles Act on Biofilm Eradication and Virulence Inhibition for Chronic Lung Infection Treatment, Journal of Controlled Release: Official Journal of the Controlled Release Society. (2024) 365, 219–235, 10.1016/j.jconrel.2023.11.032, 37992874. 37992874

[46] Narano Y. , Studies on Red Pigments Production of *Pseudomonas aeruginosa* (Strain No. 145) (Report IV), Journal of Kansai Medical University. (1967) 19, no. 2, 93–102, 10.5361/jkmu1956.19.2_93.

[47] Zhang Y. , Huang C. , Zhao M. , Li G. , Wu Z. , Zhang J. , and Zhang L. , Whole-Transcriptome Sequencing Reveals That mRNA and ncRNA Levels Correlate With *Pleurotus cornucopiae* Color Formation, Horticulturae. (2024) 10, no. 1, 10.3390/horticulturae10010060.

[48] Weiser R. , Donoghue D. , Weightman A. , and Mahenthiralingam E. , Evaluation of Five Selective Media for the Detection of *Pseudomonas aeruginosa* Using a Strain Panel From Clinical, Environmental and Industrial Sources, Journal of Microbiological Methods. (2014) 99, 8–14, 10.1016/j.mimet.2014.01.010, 24472675. 24472675

[49] Perry J. D. , A Decade of Development of Chromogenic Culture Media for Clinical Microbiology in an Era of Molecular Diagnostics, Clinical Microbiology Reviews. (2017) 30, no. 2, 449–479, 10.1128/CMR.00097-16, 28122803. PMC5355637 28122803

[50] Festus O. D. and Emmanuella O. O. , Testing the Efficacy of Mueller Hinton Agar Over Nutrient Agar for Optimal Antibiotic Sensitivity Testing Response by Selected Clinical Bacterial Pathogens, GSC Advanced Research and Reviews. (2020) 5, no. 2, 61–74, 10.30574/gscarr.2020.5.2.0037.

[51] Hombach M. , Maurer F. P. , Pfiffner T. , Böttger E. C. , and Furrer R. , Standardization of Operator-Dependent Variables Affecting Precision and Accuracy of the Disk Diffusion Method for Antibiotic Susceptibility Testing, Journal of Clinical Microbiology. (2015) 53, no. 12, 3864–3869, 10.1128/JCM.02351-15, 26468500. PMC4652116 26468500

[52] Cherkaoui A. , Renzi G. , Fischer A. , Azam N. , Schorderet D. , Vuilleumier N. , and Schrenzel J. , Comparison of the Copan WASPLab Incorporating the BioRad Expert System Against the SIRscan 2000 Automatic for Routine Antimicrobial Disc Diffusion Susceptibility Testing, Clinical Microbiology and Infection. (2020) 26, no. 5, 619–625, 10.1016/j.cmi.2019.11.008, 31733376. 31733376

[53] Matuschek E. , Brown D. F. J. , and Kahlmeter G. , Development of the EUCAST Disk Diffusion Antimicrobial Susceptibility Testing Method and Its Implementation in Routine Microbiology Laboratories, Clinical Microbiology and Infection. (2014) 20, no. 4, O255–O266, 10.1111/1469-0691.12373, 24131428. 24131428

[54] Nassar M. S. M. , Hazzah W. A. , and Bakr W. M. K. , Evaluation of Antibiotic Susceptibility Test Results: How Guilty a Laboratory Could Be?, Journal of the Egyptian Public Health Association. (2019) 94, no. 1, 10.1186/s42506-018-0006-1, 30686832. PMC6329728 30686832

